# The Effect of Gap Distance between a Pin and Water Surface on the Inactivation of *Escherichia coli* Using a Pin-to-Water Plasma

**DOI:** 10.3390/ijms23105423

**Published:** 2022-05-12

**Authors:** Junghyun Lim, Eun Jeong Hong, Seong Bong Kim, Seungmin Ryu

**Affiliations:** Institute of Plasma Technology, Korea Institute of Fusion Energy, Gunsan-si 54004, Korea; limjh@kfe.re.kr (J.L.); mealie@kfe.re.kr (E.J.H.); sbkim@kfe.re.kr (S.B.K.)

**Keywords:** pin-to-water plasma, singlet oxygen, gap distance, bactericidal effect, *Escherichia coli*

## Abstract

Atmospheric plasmas have been applied for the inactivation of microorganisms. Industrials demand to investigate the relation of the key reactive species induced by plasmas and the operating parameters including boundary conditions in order to control plasma treatment processes. In this study, we investigated the effect of gap distance between a pin-electrode and water surface on inactivation efficacy. When the gap distance decreased from 5 mm to 1 mm, the reduction of *Escherichia coli* (*E. coli*) was increased to more than 4 log CFU/mL. The reactive oxygen species measured optically and spectrophotometrically were influenced by gap distance. The results from electron spin resonance (ESR) analysis showed that the pin-to-water plasma generated hydroxyl radical (OH•) and singlet oxygen (^1^O_2_) in the water and superoxide radical (O_2_^−^•) served as a precursor of OH•. The inactivation of *E. coli* was significantly alleviated by sodium azide (^1^O_2_ scavenger), indicating that ^1^O_2_ contributes the most to bacterial inactivation. These findings provide a potentially effective strategy for bacterial inactivation using a pin-to-water plasma.

## 1. Introduction

With the fast-growing industrialization and urbanization, the demand for clean water continues to grow. Despite a great development in water disinfection techniques over the last few decades, bacterial decontamination is still a scientific and industrial challenge. At least 1006 cases of illness, 124 hospitalizations, and 13 deaths were reported as water-associated diseases in the US during 2013–2014 [1]. *Escherichia* *coli* (*E. coli*), the main species in the fecal coliform group, is regarded as one of the most common microorganism of food and wastewater [2]. In addition, *E. coli* is associated with illnesses such as nausea, vomiting, and diarrhea [3]. Chlorination has been widely used for the disinfection of such a microorganism. However, it has shown a negative effect causing the formation of disinfection by-products (DBPs) [4]. To overcome these issues, researchers have developed advanced oxidation processes (AOPs) such as ozonation, photocatalysis, sonolysis, and plasma treatment [5,6].

Atmospheric pressure plasma has shown great potential in various applications, such as water purification, biomedicine, agriculture, and the food industry [7,8,9,10,11]. In particular, it is effective for the inactivation of bacteria [12], viruses [13], and fungi [14]. The effectiveness of plasma treatment on microbial inactivation is determined by various factors such as operation mode, the type of device, and reactor design. First, the inactivation performance largely depends on the operation mode [15]. In the indirect mode, plasma-treated water (PTW) is prepared as a result of an interaction between water and plasma-initiated active species. Because PTW does not contain plasma-induced ultraviolet light and heat, its use is advantageous for the decontamination of heat-sensitive samples [9]. Meanwhile, a high microbial load or the decomposition of complex organic pollutants due to a significantly low contribution of plasma-induced short-lived species is not appropriate. In contrast, in the direct mode, the target sample or surrounding solution is exposed to the plasma discharge. Because microorganisms or organic materials directly react with the plasma-induced short-lived species, it shows high efficiency for the removal of pollutants [13]. Among plasma-induced short-lived species, short-lived reactive oxygen species (ROS), such as OH radicals (OH•), singlet oxygens (^1^O_2_), and superoxide radicals (O_2_^−^•) play the most important role in the bactericidal process. For instance, the addition of ^1^O_2_ scavenger completely suppressed virus inactivation, indicating that ^1^O_2_ has a key role in the virus inactivation test [13]. Another study showed that ^1^O_2_ and O_2_^−^• were key species in the bactericidal process. [16]. The population of *E. coli* in the O_2_^−^•, ^1^O_2_, or the peroxynitrite scavenger added group was not reduced significantly under direct current (DC)-driven air plasma. Based on the theoretical mechanism and chemical experiments, it was concluded that O_2_^−^• and ^1^O_2_ were the decomposition products of peroxynitrite, and they have a key role in microbial inactivation. OH•, a well-known antibacterial agent, was also regarded as a key species for *Candida glabrata* inactivation under DC-driven micro-discharge [17]. Second, the inactivation performance is also influenced by the type of plasma-generating device. Non-thermal plasma can be generated under corona discharge, dielectric barrier discharges (DBD), microwaves (MW), and gliding arc discharge [18]. Plasma jet and DBD are frequently used as plasma generating devices due to their ease of construction and adoption [19]. Meanwhile, the use of such devices in the industry is limited due to some drawbacks, such as low energy efficiency and high gas consumption in some plasma technologies [20,21]. The application of pin-to-water can be considered for industrial use because it has a higher energy efficiency for microbial inactivation than other plasma applications, such as DBD [22]. Similarly, pin-to-liquid plasma treatment shows higher removal efficiency of diatrizoate, present in hospital effluent, compared to DBD and wire-to-water plasma [23]. In addition, in many cases, pin-to-water plasma is a simple and low-cost system, because external gases such as argon or helium are not necessary. The pin-to-water plasma system consists of a high voltage pin electrode in the gas phase above the water surface and the ground electrode immersed in water. The plasma channels are generated from the pin electrode to the water surface generating various physical and chemical processes including UV radiation, shock waves, and numerous ROS and reactive nitrogen species (RNS). The plasma spreading over the water surface also reacts with gas and water molecules producing strong oxidizers. The radicals and molecules are injected into the water to generate secondary reactive species. Meanwhile, the production rate of ROS and RNS by DBD or plasma jet is relatively low [24]. Consequently, the concentration of nitrite (NO_2_^−^) and hydrogen peroxide (H_2_O_2_) produced by pin-to-water discharge has a higher efficiency than that made by various types of DBD [25]. Our research group has investigated pin-to-water plasma for the last few years and discovered that the characteristics of the plasma and the plasma-treated water are dependent on the gap distance between the pin electrode and the water surface. In addition, there are also many studies on the pin-to-water plasma discharge focusing on the plasma characteristics. Nevertheless, to date, the relation between the operating parameters and the antimicrobial efficacy is not well understood.

In this study, therefore, we deployed pin-to-water plasma for the inactivation of microorganisms. This study aimed to clarify the effect of the discharge time and the gap distance between the pin electrode and the water surface on the population of microorganisms. We also investigated the effect of the operating parameters on the characteristics of the plasma and the plasma-treated solution. In addition, the role of ROS was evaluated, based on a scavenger test. *E. coli* was used as the model bacteria because it is a widely used pathogenic bacteria and regarded as an indicator organism [26].

## 2. Results

### 2.1. Discharge Characteristics of the Pin-to-Water Plasma

**Figure 1:**
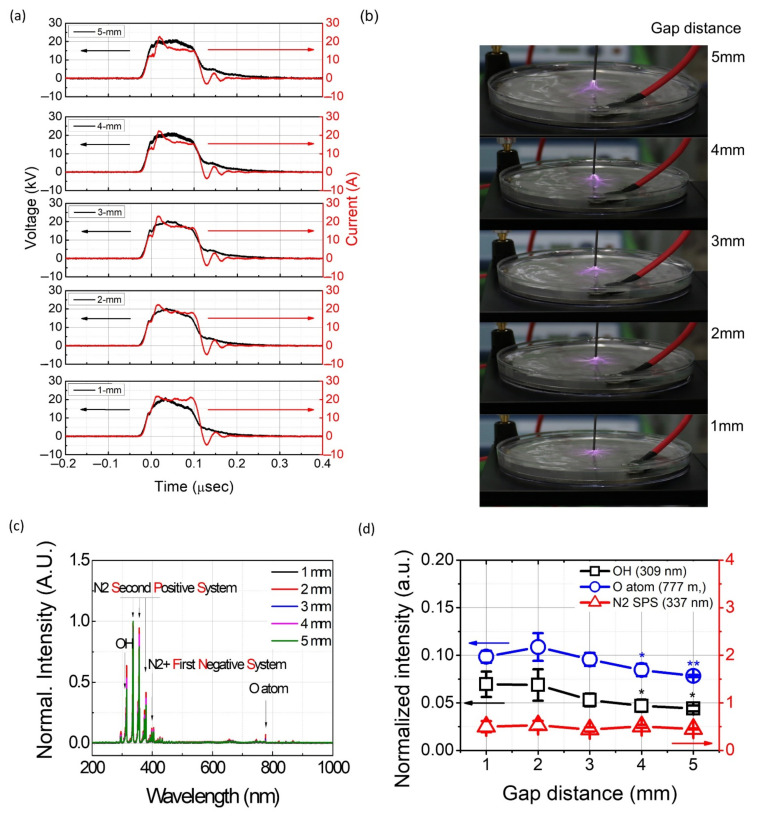
The characteristics of the plasma with different gap distances (**a**) current and voltage profile; (**b**) images of pin-to-water discharge; (**c**) OES (Optical Emission Spectra) of the plasma; and (**d**) relative intensity of N_2_ SPS (337 nm), OH (309 nm), and O atom (777 nm). Results are presented as the mean ± standard deviation from three independent experiments.

Figure 1a shows the electrical characteristics (voltage and current) measured in the discharge between the pin electrode and the water surface with different gap distances. The power settings were maintained at a 100-nanosecond pulse width and 2 kHz frequency. The breakdown occurred when the peak voltage was about 22.5 kV, and the peak current was about 22 A. In all cases, the voltage waveforms were relatively similar. After the breakdown, the current remained almost constant for 100 nanoseconds under the plasma discharge with a 1-mm gap distance. Meanwhile, it was observed that the current continuously decreased after the breakdown with a 5-mm gap indicating that the current was influenced by the gap distance during the plasma discharge. These results might be derived from the air gap which acts as resistance. Based on the I-V curve, the averaged discharge power was calculated by Equation (1) as follows [21]:

(1) $$P\left(W\right)=\frac{1}{T}\int_{0}^{T}U\left(t\right)\times I\left(t\right)\times dt$$

where *P*(*W*) is the output power; *U*(*t*) and *I*(*t*) are the instantaneous current and voltage at time *t*; and *T* is the period of voltage. When the gap distance was 1 mm, the discharge power was 68.8 ± 1.0 W. Meanwhile, the discharge power was dependent on the gap distance. When the gap distance was 2, 3, 4, and 5 mm, it was 68.6 ± 1.0, 67.2 ± 1.6, 64.8 ± 1.3, and 59.5 ± 2.1 W, respectively, based on each I-V curve.

Different electrical characteristics may influence the plasma behavior, as shown in Figure 1b. At a 1-mm gap, much filament-like plasma propagates at the water surface. In contrast, the number and length of these phenomena were decreased when the gap was increased to 5 mm. To understand the plasma characteristics induced by the different gap distances, optical emission spectra (OES) were used with the different gaps, and they presented overlapped optical emission signals, shown in Figure 1c. Note that the OES spectra shown in Figure 1c were normalized to the maximum values for each gap distance [27]. In all cases, a strong N_2_ second positive system (SPS) (C^3^Π_g_→B^3^Π_g_) at 315.8, 337, 357, and 380, and N_2_^+^ first negative system (FNS) (B^2^Σ_u_^+^→X^2^Σ_g_^+^) at 391 nm were found to be the dominant emission spectra. Atomic oxygen lines (777 and 844 nm) and a small amount of an OH line (309 nm) were also observed. Figure 1d shows the intensity variation of the 309 nm OH radical, 337 mm N_2_ SPS, and 777 O atom for the various gap distances, according to a previously introduced method [28]. Our results show that the intensity of N_2_ SPS did not change in all of the treatment conditions (*p* > 0.05). The intensity of the 309 nm OH∙ and 777 O atom decreased as the gap distance was increased. In particular, significant differences were observed under 4 mm or longer gap distances compared to the 1 mm gap (*p* < 0.05).

### 2.2. Changes in the Properties of the Plasma-Treated Liquid

**Figure 2:**
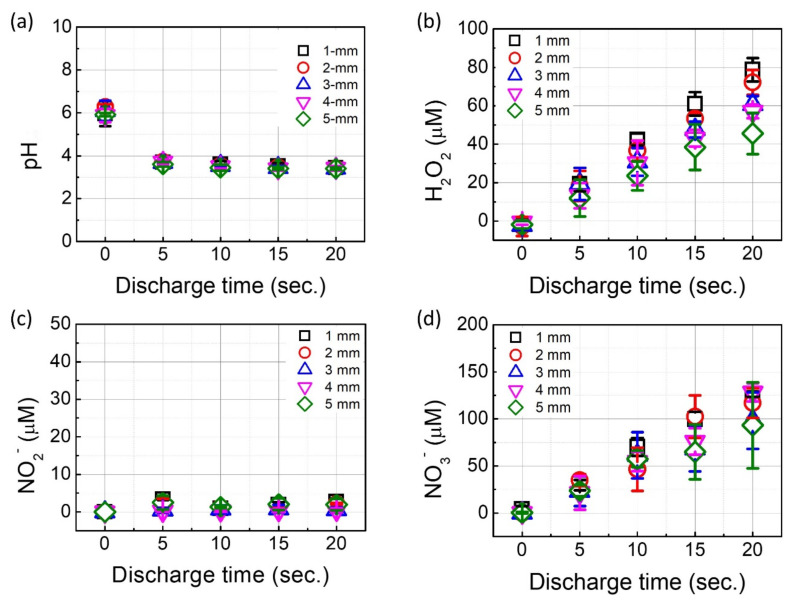
The characteristics of the plasma-treated liquid after different plasma treatment times and gap distances; (**a**) pH; (**b**) H_2_O_2_; (**c**) NO_2_^−^; and (**d**) NO_3_^−^. Results are presented as the mean ± standard deviation from three independent experiments.

Chemical analysis was conducted during a plasma discharge for 20 s with different gap distances, and the results are shown in Figure 2. As seen in Figure 2a, the pH of the solution decreased during the 20-s plasma treatment. The initial pH value was around 5.71, and the pH decreased to around 3.69, indicating that acidification could occur under the plasma discharge. Nevertheless, our results show that the pH value was not influenced by the gap distance. Likewise, no significant relationship was found between the gap distance and the concentration of RNS. The concentration of NO_2_^−^ did not change during the plasma discharge, irrespective of the different gap distances. Although the nitrate (NO_3_^−^) concentration was increased with the plasma discharge time, the gap distance did not show any significant difference (*p* > 0.05). On the other hand, the gap distance had an impact on the concentration of H_2_O_2_ (Figure 2b). After a 20-s treatment, the H_2_O_2_ concentrations obtained were 78.7 ± 6.06, 72.2 ± 6.49, 60.6 ± 4.45, 57.1 ± 3.56, and 45.6 ± 10.7 μM with a 1, 2, 3, 4, and 5-mm gap distance, respectively. In particular, the result with a 5-mm gap distance was significantly different from the other experimental conditions (*p* < 0.05).

### 2.3. The Effect of the Operating Parameters on the Population of E. coli

**Figure 3:**
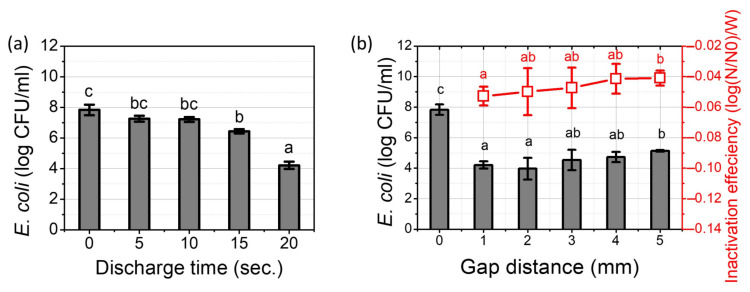
Surviving population (log (CFU)/mL) of *E. coli* after different treatment conditions; (**a**) different plasma treatment times (0, 5, 10, 15 and 20 s) with a 1-mm gap distance; (**b**) different gap distances with a 20-s treatment time (gray bars), and inactivation of the *E. coli* population per input power in watt (red dots). Results are presented as the mean ± standard deviation from three independent experiments. Different letters indicate significant differences (*p* < 0.05).

The inactivation efficacy of the pin-to-water plasma treatment against an *E. coli* suspension was monitored as a function of the discharge time and gap distance. The effect of the plasma treatment time of the pin-to-water plasma on the population of *E. coli* was conducted for 20-s with a 1-mm gap distance, as shown in Figure 3a. The number of viable cells was decreased significantly in proportion with the treatment time. The 5-s plasma treatment had a significantly reduced *E. coli* population (*p* < 0.05), and a 4-log reduction was achieved by the 20-s treatment.

Figure 3b (gray bars) shows that the amount of surviving population was related to the gap distance. A significant reduction of the microbial population was observed after a 20-s plasma treatment compared to the control, irrespective of the various gap distances. Nevertheless, bactericidal efficacy seems to be dependent on the gap distance. When the gap distance was 1 mm, a 4.21 log reduction was observed after a 20-s treatment. Meanwhile, 3.41, 3.16, and 2.68 log reductions were observed when the gap distance was 2, 3, 4, and 5 mm, respectively. In particular, the number of surviving *E. coli* by the plasma treatment with a 1 mm gap distance significantly differed from the values obtained for 5 mm (*p* < 0.05). An input power-based analysis was conducted with different gap distances according to Equation (2), and the results are shown in Figure 3b (red dots):

(2) $$\frac{\left({\mathrm{Bacterial}\mathrm{count}}_{control}-{\mathrm{Bacterial}\mathrm{count}}_{plasma\;treated\;with\;different\;gap}\right)}{Input\;power\;\left(W\right)}$$

Our results show that the plasma treatment with a shorter gap was effective and efficient. Approximately a 0.053 log reduction per 1 W was observed with a 1-mm gap, while a 0.049, 0.047, 0.041, and 0.040 log reduction was observed with the 2, 3, 4, and 5-mm gaps, respectively.

### 2.4. Electron Spin Resonance Spectroscopy Study

**Figure 4:**
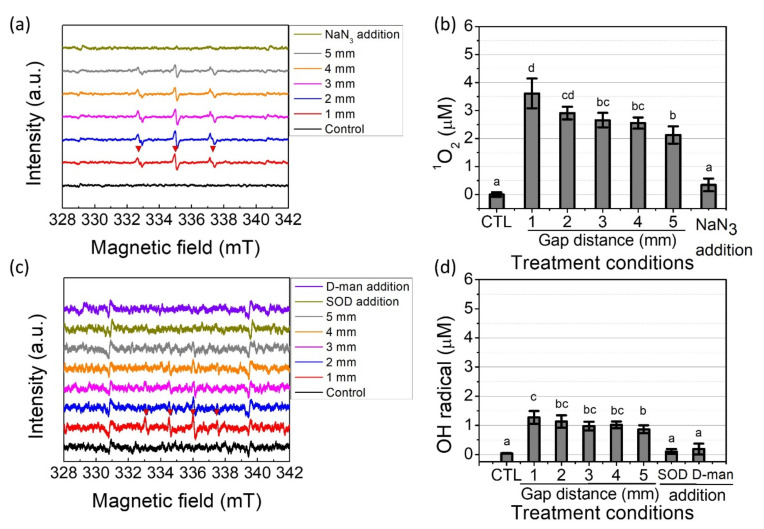
Electron spin resonance signals and concentration from pin-to-water plasma treatment (**a**) TEMPO (spin adduct of ^1^O_2_); (**b**) concentration of ^1^O_2_; (**c**) DMPO-OH (spin adduct of OH•); (**d**) concentration of OH•. The treatment time was kept at 20 s. Results are presented as the mean ± standard deviation from three independent experiments. Different letters indicate significant differences (*p* < 0.05).

To study the role of short-lived ROS in PTW on the bactericidal effect, an ESR spectroscopy was used to identify whether representative ROS (^1^O_2_, OH•, and O_2_^−^•) were produced during the plasma treatment. In addition, the scavenger test was conducted to determine whether each short-lived ROS was completely suppressed. Due to the very short lifetime of the ROS, spin trapping methods were carried out using 5,5-dimethyl-1-pyrroline N-oxide (DMPO) and 2,2,6,6-Tetramethylpiperidine (TEMP). Figure 4a shows the three peak spectrum with an intensity ratio of 1:1:1, and the hyperfine coupling constants were a_N_ = 17 G, which are consistent with the signals for 2,2,6,6-Tetramethylpiperidine-1-oxyl (TEMPO) [29]. Because TEMPO signals are generated by the spin trapping reaction between TEMP and ^1^O_2_, the formation of ^1^O_2_ was identified. The intensity was influenced by the gas distance and the addition of a scavenger. For the quantitative analysis of ^1^O_2_, a calibration curve was made using a standard TEMPO solution, as shown in Figure S1 (Supplementary Materials) [30]. The concentration of ^1^O_2_ was dramatically increased by the 20-s plasma discharge, and the concentration was influenced by the gap distance: 3.6 ± 0.5; 2.9 ± 0.2; 2.6 ± 0.3; 2.5 ± 0.2; and 2.1 ± 0.3 μM, with a gap distance of 1, 2, 3, 4, and 5 mm, respectively. After the 9 mM sodium azide (NaN_3_) was added to the TEMP solution, the signal disappeared (dark yellow line), indicating that the amount of NaN_3_ was enough to eliminate the formation of ^1^O_2_. Take note that the concentration of TEMP was not so high as to trap all the produced ^1^O_2_ because too much TEMP addition causes an increment of the conductivity and changes in the plasma characteristics. Thus, the actual concentration of ^1^O_2_ can be higher than presented in this study. Because NaN_3_ reacts with not only the ^1^O_2_ (rate constant: 2.2 × 10^9^ M^−1^s^−1^) but also with the OH• (rate constant: 1.1 × 10^10^ M^−1^s^−1^), it is hard to expect the presence of ^1^O_2_ and OH• in the plasma-treated NaN_3_ solution [31].

In the presence of aqueous OH•, DMPO can trap OH•, forming DMPO-OH. As shown in Figure 4c, plasma generation produces the spin adduct of DMPO-OH with four lines, an intensity ratio of 1:2:2:1, and hyperfine coupling constants a_N_ = a_H_ = 14.9 G (red line) [32]. Meanwhile, the intensity decreased as the gap distance was increased. It is hard to quantify the amount of OH• because there is no standard solution for the comparison. Nevertheless, roughly quantifying the aqueous OH• was approached by using TEMPO as a standard solution, according to the previous method [32]. Figure 4d shows that the concentration of OH• was around 1.27 μM under a 1-mm gap which is significantly higher than that of the treated one with a 5-mm gap. It is essential to know that the DMPO-OH signal originates from two different scenarios: a direct reaction between DMPO and OH• and a reaction between DMPO and OOH• producing DMPO-OOH, which further decomposes into DMPO-OH due to a short half-life of 1 min [33]. Thus, the origin of DMPO-OH was determined with different scavengers (D-mannitol and superoxide dismutase (SOD)) to analyze the plasma-induced liquid chemistry. Therefore, 10 mM D-mannitol was added before the plasma discharge to evaluate the amount of the added OH• scavenger and to determine whether DMPO-OH was a product of the reaction between DMPO and OH•. It was found that the DMPO-OH signal disappeared by the D-mannitol addition, suggesting that DMPO-OH was produced directly from OH•, and the 10 mM D-mannitol was enough to eliminate the OH• (dark yellow line). Meanwhile, the DMPO solution containing SOD did not show any signal (purple line), indicating that O_2_^−^• was converted into OH•. Thus, it was concluded that OH• was produced from the decomposition of O_2_^−^•. The concentration of OH• in the SOD and D-mannitol added groups had less than 0.2 μM, which was not significantly different from the control group.

### 2.5. The Role of Different ROS in the Plasma Inactivation of E. coli

**Figure 5:**
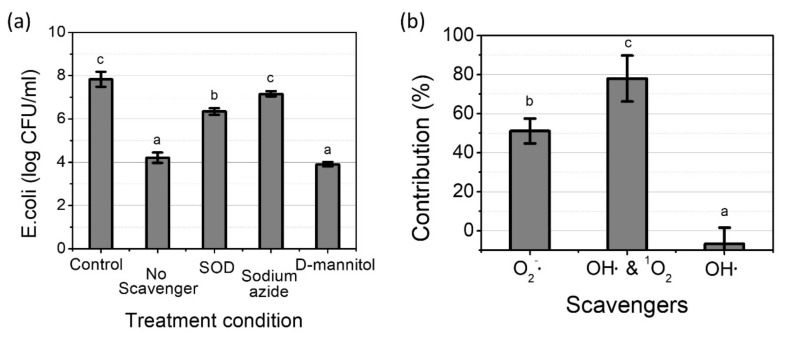
The effect of scavengers on *E. coli* after a plasma treatment with a 1-mm gap; (**a**) surviving *E. coli* population with different scavengers; (**b**) contribution ratio of each short-lived species. Results are presented as the mean ± standard deviation from three independent experiments. Different letters indicate significant differences (*p* < 0.05).

In order to investigate the role of ROS on the inactivation rate of *E. coli*, scavenger tests were conducted with a 1-mm gap distance. Three ROS scavengers, which were D-mannitol for OH•, SOD for O_2_^−^•, and sodium azide for OH• and ^1^O_2_, were used to distinguish the roles of the different short-lived species on the inactivation by the pin-to-water plasma discharge time. As shown in Figure 5a, the surviving population of *E. coli* was significantly decreased from 7.83 ± 0.34 log/mL to 4.21 ± 0.24 log/mL with the plasma treatment times ranging from 0 to 20 s. The survival rate in the 10 mM D-mannitol added group was not significantly different from the no scavenger added group (*p* > 0.05), indicating that the OH• generated by the pin-to-water plasma contributes little to the *E. coli* inactivation. In contrast, the inactivation rate was significantly alleviated in the presence of sodium azide and SOD, compared to the untreated condition. During the 20 s treatment, only 0.67 and 1.48 log reductions were found in the sodium azide and SOD added groups. The influence of each short-lived ROS was presented as the contribution ratio, which is defined as the proportion of the decreased population of *E. coli* by a scavenger containing solution to the overall decreased number of *E. coli* after the plasma treatment. The contribution ratio was calculated with Equations (3)–(5), according to previous research with a slight modification, and the results are presented in Figure 5b [34]:

(3) $$\mathrm{Contribution}\left(\%\right)=\frac{r}{R}\times 100$$

(4) $$r=-\log\left(\frac{S_{20}}{S_{0}}\right)$$

(5) $$R=-\log\left(\frac{W_{20}}{W_{0}}\right)$$

where, *S*_0_ is the number of untreated *E. coli* in the scavenger (SOD, sodium azide, or D-mannitol) containing solution, while *W*_0_ is the number of untreated *E. coli* in water. *S*_20_ is the number of *E. coli* in the scavenger containing solution after a 20-s plasma treatment with a 1-mm gap, while *W*_20_ is the number of *E. coli* in water after a 20-s plasma treatment with a 1-mm gap.

The contribution ratios of SOD, sodium azide, and D-mannitol were 51.1 ± 6.4%, 77.9 ± 11.8%, −6.8 ± 8.3%, respectively. Since the contribution of OH• was negligible in this study, the influence of sodium azide mostly originated from ^1^O_2_. Consequently, our result shows that ^1^O_2_ contributes the most to bacterial inactivation.

## 3. Discussion

Based on the inactivation test, it is clear that pin-to-water plasma treatment is an effective method for the removal of microorganisms. It was shown that 99.99% reduction of *E. coli* was achieved by the 20 s treatment. The energy cost to obtain 1 log reduction was approximately 9 kJ/L, which was more efficient than the nano- and micro-pulsed electric field (100–158 kJ/L) [35], but less than UV-C treatment [36]. As the plasma system is easy to set-up and use, it is feasible to consider the use of pin-to-water plasma for the removal of microorganisms in aqueous phase.

To clarify the microbial inactivation mechanism induced by plasma treatment, the characteristics of plasma and plasma-treated water were investigated with different gap distances. The characteristics of plasma resulting from the optical emission spectrum have reveal that only the ROS level was influenced by gap distance. As the intensity of the OH and O emissions could be related to the density of the water vapor, the OH and O emissions could be caused by the reaction between the electron and water vapor by the high plasma energy through various reaction pathways ($e+H_{2}O\to H+OH+e;\;e+H_{2}O\to H_{2}^{+}+O+2e$) [5,37]. Since the emission intensity can be determined by various factors including gas temperature, electron temperature, electron density, the density of excited species, and the quenching rate, it is hard to infer the direct relationship between the optical emission intensity and the concentration of reactive species in the aqueous phase. Nevertheless, qualitative trends could be considered. For instance, a strong correlation has been suggested between the production of RONS in plasma and solution [38]. Our experiments also show that only the concentration of ROS such as H_2_O_2_, ^1^O_2_, and OH• in water was associated with the gap distance. In general, the H_2_O_2_ can be made from the reaction of two OH∙ through reaction (6) [39]. Alternately, when the density of OH∙ is too low, H_2_O_2_ could also be generated from O_2_^−^• in an acidic condition (pH < 4.8) through reaction (7) [40]:

(6) $$\mathrm{OH}\cdot +\mathrm{OH}\cdot \to H_{2}O_{2}$$

(7) HO2·+HO2· →H2O2+O12

Meanwhile, no significant difference in the concentration of RNS was caused by the different gap distance. The nitric oxide was produced by the dissociation of N_2_ and O_2_ during the plasma discharge with reaction (8). A portion of nitric oxide can react with oxygen to produce nitrogen dioxide (reaction (9)), and the dissolution of nitric oxide and nitrogen dioxide were the origin of both NO_2_^−^ and NO_3_^−^ with reaction (10) and (11) [39,41]. As the density of nitrogen and oxygen was expected to be much higher than other molecules, their concentration did not seem to depend on the gap distance. The gap distance did not have an influence on pH values (Figure 2a) as the hydrogen ions released during RNS generation were not significantly different (Equations (10) and (11)):

(8) $$N_{2\left(g\right)}+O_{2\left(g\right)}\;\overset{plasma}{\to }2NO$$

(9) $$2NO+O_{2\left(g\right)}\to \;NO_{2}{}_{\left(g\right)}$$

(10) $$NO_{2\left(g\right)}+NO_{2\left(g\right)}+H_{2}O_{\left(l\right)}\;\to \;NO_{2}^{-}+NO_{3}^{-}+2H^{+}$$

(11) $$NO_{\left(g\right)}+NO_{2\left(g\right)}+H_{2}O_{\left(l\right)}\;\to \;2NO_{2}^{-}+2H^{+}$$

To further investigate the role of ROS in the inactivation of *E. coli*, a scavenger test was conducted. Three major ROS that are frequently reported as a bactericidal agents, ^1^O_2_, O_2_^−^•, and OH•, were selected to investigate the role of ROS in the microbial inactivation mechanism [42,43]. Despite a short lifetime of 10^−9^ s, OH• is regarded as one of the most powerful antibacterial agents, due to its high oxidation potential of 2.8 eV. In particular, OH• has a very low CT value of 1.5 × 10^−5^ mg/liter × min for a 2-log inactivation of *E. coli* [44]. Nevertheless, OH• induced by plasma have shown very low inactivation efficiencies. For instance, approximately 12 and 0.6 μM of OH• generated by DBD plasma did not show an important role in the inactivation of viruses and bacteria [13,32]. It is possible to infer that there is a deactivating ability of OH• in plasma-treated water. On the contrary, ^1^O_2_ contributes the most to the bacterial inactivation, in spite of a lower inactivation ability (1.3 × 10^−4^ mg/liter × min for 2-log inactivation of *E. coli*) than that of OH• [45]. It is possibly due to better diffusion among biological membranes and its high concentration and, relatively, long lifetime of 10^−5^ s [46]. Furthermore, ^1^O_2_ could be a more effective oxidant than OH• for practical applications, because it is less likely to be affected by aqueous inhibitors such as natural organic matter [47]. Meanwhile, no significant reduction was observed in the sodium azide added solution indicating that the *E. coli* count was not significantly influenced by physical parameters, such as shock waves or UV radiation. Although O_2_^−^• is not regarded as highly reactive species, the contribution of O_2_^−^• was not negligible. When the pH is lower than a pKa of 4.8, a lot of O_2_^−^• are converted to hydroperoxy radicals (HO_2_•), which can penetrate the cell membrane and attack cytosolic targets [48]. As the pH value of the plasma-treated solution was around 4 (Figure 3a), almost O_2_^−^• must be converted to HO_2_•. Because the gap distance does not have an impact on the RNS level but on the ROS level, it is feasible to infer that the inactivation process seems to have some relationship with the ROS. In our experiment, ^1^O_2_ contributes the most to the bacterial inactivation, indicating that ^1^O_2_ generating device are essential to increase energy efficiency. These species can attack microorganisms resulting in lipid peroxidation, and release of protein and nucleic acid [49]. Alternately, they can be converted to other species due to their instability [50]

The atmospheric pressure plasma treatment is a good alternative to other conventional methods, including autoclaving, dry heat, gamma ray, and UV irradiation, since it has a number of advantages, such as long operation time, mild temperature, no toxic residues, and a simple system [51]. Nevertheless, higher energy efficiency is an important challenge for the industrial use. Our results show that singlet oxygen is a key species in the bactericidal process, and it is necessary for more singlet oxygen generation plasma systems to be developed.

## 4. Materials and Methods

### 4.1. Chemicals

Chemical scavengers for reactive species, D-mannitol (scavenger of hydroxyl radicals (OH•)), sodium azide (scavenger of singlet oxygens (^1^O_2_)), and SOD (scavenger of superoxide radicals (O_2_^−^•)), were purchased from Sigma-Aldrich, St. Louis, MO, USA. Spin trap solutions, including TEMP and TEMPO, were purchased from Sigma-Aldrich, St. Louis, MO, USA. DMPO was purchased from Tokyo Chemical Industry, Tokyo, Japan. The hydrogen peroxide kit used for the determination of aqueous hydrogen peroxide (H_2_O_2_) was obtained from Humas, Daejeon, Korea.

### 4.2. Preparation of E. coli Suspension

The *E. coli* (KCTC 2441) used in this study was obtained from the Korea Collection for Type Culture, Jeongeup, Korea. A fresh culture of *E. coli* was cultivated in fresh sterile Luria-Bertani broth (LB, Difco, Sparks, MD, USA) at 150 rpm and 37 °C for 24 h (SI- 300R, Jeio Tech Co., Ltd., Deajeon, Korea). For the removal of organic matter, the medium was washed three times with deionized water (DW) by centrifugation at 5000× *g* for 10 min at 4 °C (Eppendorf 5810 R, Hamburg, Germany). The final pellets were re-suspended in DW, and the final concentration of *E. coli* was approximately 5 × 10^7^ cells/mL.

### 4.3. Plasma Device

**Figure 6:**
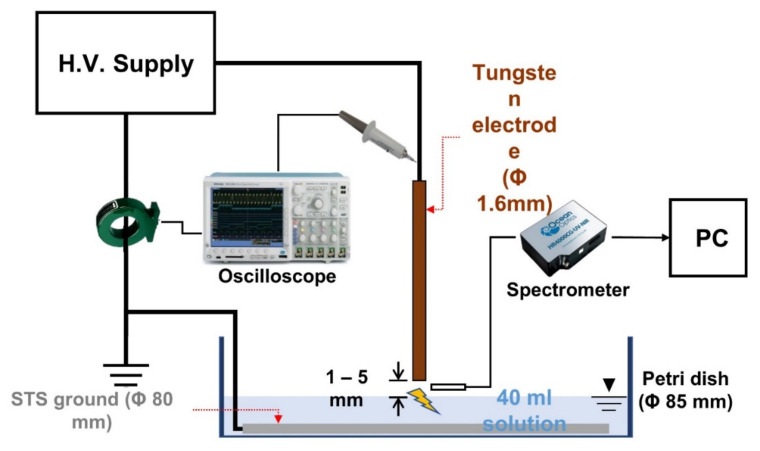
The schematic diagram of the plasma device.

The schematic diagram of the plasma device is shown in Figure 6. The structure of the pin-to-water system consists of two parts: a high-voltage tungsten electrode (Φ 1.6 mm), and a stainless-steel ground electrode plate (Φ 80 mm) on a petri dish (Φ 85 mm, 2.3 mm thickness). A nanosecond pulsed power supply (NSP-5000, Eagle Harbor Technologies, LLC, Seattle, WA, USA) was connected to the pin electrode and STS ground electrode. The power settings used in this study were as follows: 100 ns pulse width and 2 kHz frequency. Forty milliliters of solution were transferred to a petri dish (10,090, SPL life science, Pocheon, Korea) before the plasma treatment.

### 4.4. Analysis of the Plasma Characteristics

Applied voltage and current profile during plasma discharge were measured by a high voltage probe (P6015A Tektronix, Beaverton, OR, USA) and fast current monitor (6585 Pearson Electronics, Palo Alto, CA, USA), respectively. The two signals were recorded using an oscilloscope (Tektronix, DPO7254). The plasma behavior was recorded using a digital camera (EOS-7D Mark II, Cannon, Tokyo, Japan). The image was taken under the following conditions: f-number 3.5, iso of 400, and exposure time of 1/200 s. The emissions characteristic of the plasma was measured using spectroscopy (HR4000CG-UV-NIR, Ocean Optics, Orlando, FL, USA) with an optical cable (Ocean Optics/QP400-2-SR). To collect the spectra during discharge, an optical lens was placed perpendicularly to the pin axis. Spectra were recorded at an integration time of 1 s and wavelength range of 200 to 1000 nm.

### 4.5. Plasma Treatment

For the microbial inactivation test, three experiments were conducted with the following procedure:

(i) To understand the effect of the gap distance between the pin electrode and liquid, gap distances were set to 1, 2, 3, 4, and 5 mm, and the plasma discharge time was fixed at 20 s;
(ii) The effect of the treatment time on the inactivation of *E. coli* was studied. In this experiment, the gap distance was kept at 1 mm. The treatment times were 0 (control), 5, 10, 15, and 20 s;
(iii) To determine the role of each reactive oxygen species on the inactivation of *E. coli*, 10 mM mannitol, 120 kU/l SOD, or 9 mM sodium azide was added to the liquid containing the *E. coli* before the plasma treatment. Each liquid (with or without scavengers) was treated with plasma for 20 s. Note that the water conductivity is not changed significantly by the added chemicals due to the low amount of scavenger.

After each experiment was finished, the plasma-treated liquid was mixed well for 30 s, and the well-mixed liquids were analyzed immediately for each purpose.

### 4.6. ESR Spin-Trapping Spectroscopy

ESR was used to identify aqueous short-lived reactive species generated by the pin-to-water plasma. For all of the measurements, the instrumental settings were as follows: central field, 336 mT; sweep width, 7.5 mT; microwave frequency, 9410 MHz; modulation frequency, 100 kHz; microwave power, 1 mW; scanning time, 30 s; and the number of scans, 3. All signals were normalized using the Mn marker (intensity: 600). Because the half-life of the short-lived reactive species was too short to measure, the spin trapping method was used. A final concentration of 100 mM DMPO and 2 mM TEMP was used to spin trap OH∙ and ^1^O_2_, respectively. Before the plasma treatment, each spin trap solution was added to deionized water. After the plasma treatment, the plasma-treated solution was measured within 1 min with an ESR spectrometer operated at room temperature. Hyperfine coupling constants of the signal were analyzed using isotropic simulation software (Jeol Ltd., Tokyo, Japan) [52].

### 4.7. Physicochemical and Chemical Analysis for Plasma-Treated Solutions

The pH, conductivity, and concentrations of H_2_O_2_, NO_2_^−^, and NO_3_^−^ of the plasma-treated solution from each experiment were determined immediately. All analytical methods were used based on the previously introduced method [53] with a slight modification. The pH was measured using a benchtop meter (Versa Star Pro^TM^, Thermo Scientific, Waltham, MA, USA) equipped with a pH probe (Orion 8157BNUMD, Thermo Scientific, USA). The concentration of H_2_O_2_ was determined using the titanium sulfate method. The color difference was measured using a UV-Vis spectrophotometer (HS-3300, Humas, Daejeon, Korea), according to the manufacturer’s instructions. NO_2_^−^ and NO_3_^−^ concentrations were measured by ion chromatography (Dionex ICS- 2100, Thermo, Sunnyvale, CA, USA) equipped with IonpacAG25 (4 × 50 mm) with 36 mM KOH as the eluent (flow rate: 1 mL/min).

### 4.8. Microbiological Analysis

A colony count assay was used to quantify the antibacterial effect of the plasma treatment. After each plasma treatment, the treated sample was immediately diluted 10-fold with a NaCl solution (0.9%). Then, 1 mL solution was spread onto 3M Petrifilm (Petrifilm^TM^ EC, 3M Company, St. Paul, MN, USA), according to the manufacturer’s instruction. Then, the Petri-film was incubated at 37 °C for two days, and the number of colonies was counted. The bacterial counts were expressed as colony forming unit (CFU) per 1 mL

### 4.9. Statistical Analysis

Data were obtained from at least three independent experiments. The values from all experiments were expressed as the mean ± standard deviation. All data were subjected to analysis of variance (ANOVA) and Student’s *t*-test, using SPSS statistics 24 software (SPSS inc., Chicago, IL, USA). Duncan’s multiple range test was used to evaluate the significance of the differences (*p* < 0.05).

## 5. Conclusions

In this study, we have shown that pin-to-water plasma is an effective method for the inactivation of *E. coli* in a planktonic state. Electrical, optical, colorimetric, biological, and ESR-based diagnostic techniques were employed to determine the characteristics of the plasma with different gap distances. More than a 4-log reduction of *E. coli* was found in the 20-s plasma treatment. Plasma discharges with a short gap of (1 mm) distance achieved higher inactivation rates and efficiency compared to a long gap (5 mm). Based on the optical analysis, more ROS production was found with a short gap distance, while the gap distance did not influence the aqueous RNS concentration. Similar trends were found in the concentration of ROS and RNS in water. The ESR study shows that ^1^O_2_ and OH• were generated during plasma discharge, and the addition of sodium azide, D-mannitol, and SOD successfully inhibited the short-lived ROS in the aqueous phase. Bacterial inactivation was significantly alleviated by the sodium azide addition, indicating that ^1^O_2_ contributes the most to the bacterial inactivation.

## Data Availability

Not applicable.

## Supplementary Materials

The following supporting information can be downloaded at: www.mdpi.com/article/10.3390/ijms23105423/s1.
