# Supplementary material for: The Effect of Gap Distance between a Pin and Water Surface on the Inactivation of Escherichia coli Using a Pin-to-Water Plasma
Source: Int J Mol Sci. 2022 May 12;23(10):5423. doi: 10.3390/ijms23105423 (PMC9145933; doi:10.3390/ijms23105423)
Supplement: Supplementary file 1 [file ijms-23-05423-s001.zip › ijms-1709792-supplementary.pdf]

APPENDIX 1. Supplementary material

The Effect of Gap Distance between a Pin and water surface on the Inactivation of *Escherichia coli* using a pin-to-water plasma

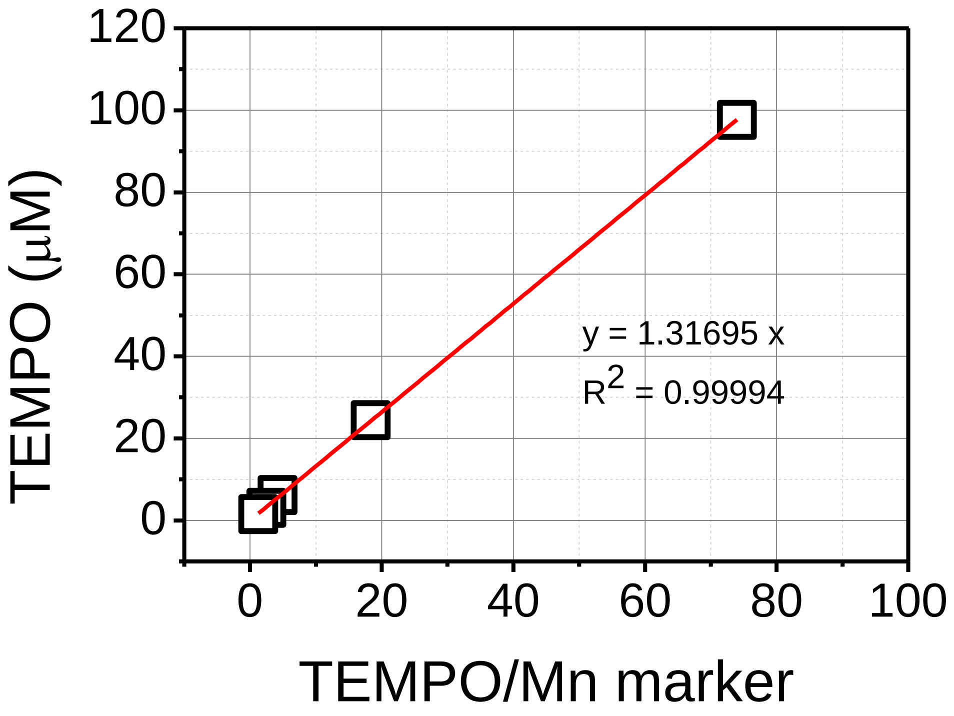

Figure S1. Calibration curve for the double integrated signal of TEMPO and the concentration of TEMPO solution.
